# Cluster Analysis of Physical Activity Patterns, and Relationship with Sedentary Behavior and Healthy Lifestyles in Prepubertal Children: Genobox Cohort

**DOI:** 10.3390/nu12051288

**Published:** 2020-05-01

**Authors:** Rosaura Leis, Jose Manuel Jurado-Castro, Francisco Jesus Llorente-Cantarero, Augusto Anguita-Ruiz, Azahara Iris Rupérez, Juan Jose Bedoya-Carpente, Rocío Vázquez-Cobela, Concepción María Aguilera, Gloria Bueno, Mercedes Gil-Campos

**Affiliations:** 1 Unidad de Investigación en Nutrición, Crecimiento y Desarrollo Humano de Galicia, Departamento de Pediatría, Hospital Clínico Universitario de Santiago, Universidad de Santiago de Compostela, IDIS, 15706 Santiago de Compostela, Spain; mariarosaura.leis@usc.es (R.L.); xanbecar@hotmail.com (J.J.B.-C.); cobela.rocio@gmail.com (R.V.-C.); 2 CIBEROBN, (Physiopathology of Obesity and Nutrition) Institute of Health Carlos III (ISCIII), 28029 Madrid, Spain; llorentefj@yahoo.es (F.J.L.-C.); augustoanguitaruiz@gmail.com (A.A.-R.); caguiler@ugr.es (C.M.A.); mercedes_gil_campos@yahoo.es (M.G.-C.); 3 Metabolism and Investigation Unit, Reina Sofia University Hospital, Maimónides Institute of Biomedicine Research of Córdoba (IMIBIC), University of Córdoba, 14071 Córdoba, Spain; juradox@gmail.com; 4 Department of Artistic and Corporal Education, Faculty of Education, University of Córdoba, 14004 Córdoba, Spain; 5 Department of Biochemistry and Molecular Biology II, Institute of Nutrition and Food Technology “José Mataix”, Center of Biomedical Research, University of Granada, Armilla, 18016 Granada, Spain; 6 Instituto de Investigación Biosanitaria ibs, 18014 Granada, Spain; 7 GENUD Research Group, University of Zaragoza, Instituto Agroalimentario de Aragón (IA2), Instituto de Investigación Sanitaria (IIS) Aragón, 50013 Zaragoza; Spain; airuperez@unizar.es; 8 Unidad de Endocrinología Pediátrica, Hospital Clínico Lozano Blesa, Facultad de Medicina, Universidad de Zaragoza, 50009 Zaragoza, Spain

**Keywords:** exercise, obesity, Mediterranean diet, cardiovascular diseases

## Abstract

Sedentary habits during childhood are associated with adverse health outcomes. The aim of this work was to cluster lifestyle behaviors and metabolic biomarkers to establish different patterns in children. Their physical and sedentary activities were evaluated by accelerometry, and questionnaires that included lifestyle behaviors, such as adherence to a Mediterranean diet, anthropometry and blood biochemical markers. Cluster analysis was performed to establish different groups based on physical activity levels. A total of 489 children were finally selected. Cluster 1 included children with a mostly sedentary state, whereas Cluster 3 included the most active children and Cluster 2 included children that did not fit into either the sedentary or the highly active groups. In Cluster 3, 56% of children were in a sports club, and a lower percentage used electronic devices in their rooms compared to the other groups. Cluster 1 children exhibited higher insulin, HOMA-IR and triacylglycerides with respect to the other groups. No differences were found regarding adherence to a Mediterranean diet. The choice to practice an extracurricular sport could be an influencing factor to increase exercise and ensure an active lifestyle in children. Reducing or limiting screen time mainly in children’s rooms could contribute to an active lifestyle.

## 1. Introduction

Obesity is increasing in an uncontrolled and continuously accelerating manner in developed societies and is currently being viewed as a pandemic. Childhood obesity, in particular, is increasing in prevalence [1], with the main concern lying not only in this increase, but also in the prevention or control of the associated metabolic and cardiovascular diseases beginning from childhood [2]. 

A sedentary lifestyle is recognized as one of the main risk factors for mortality due to non-communicable diseases, such as obesity [3]. Children and adolescents spend more time in sedentary activities than a decade ago, possibly due to the rise in electronic screen devices and the wide accessibility to the Internet [4], alongside other aspects such as lifestyle or eating habits [5,6].

Scientific evidence shows that regular physical activity (PA) provides essential benefits for children’s health, improving cardiorespiratory fitness, muscle function, bone health, and reducing body fat [7], as well as reducing the risk of developing cardiovascular diseases, diabetes or obesity [8]. Furthermore, these metabolic state benefits are greater with moderate to vigorous physical activity (MVPA), thereby increasing quality of life in children [7,9].

Growth stages are key periods in the establishment of healthy behavior patterns to avoid obesogenic and sedentary habits that could have a negative impact on future health [10,11]. To fight against this pandemic, different intervention programs focused on increasing PA and reducing sedentary activity have been carried out in children [12,13,14]. However, the aspects that influence lifestyle or increase the risk of obesity were shown to be different [15]; thus, it is necessary to understand how different patterns of lifestyle, diet, and objectively measured PA interfere with each other [7].

Therefore, the aim of this study was to identify different PA and sedentary patterns in children and to identify associations with other habits related to screen time, sleep time, diet quality, sport, and metabolic status.

## 2. Materials and Methods

### 2.1. Study Population

A total of 687 children participated in the GENOBOX study, which was carried out in three Spanish cities: Santiago de Compostela, Zaragoza and Córdoba. We recruited children attending the hospital for diagnosis of minor disorders; that were not confirmed after clinical and laboratory investigations, or suspecting overweight or obesity. After, they were invited to participate in the study. For the current study, a subsample of children was randomly selected using the following inclusion criteria: Children in good health and normal weight, overweight, and with obesity, 5–14 years of age, absence of endogenous obesity, and having a minimal amount of useful accelerometer data. Exclusion criteria were disease or malnutrition and the use of medications that altered blood pressure, glucose, or lipid metabolism, or not meeting the inclusion criteria.

The study was conducted in accordance with the Declaration of Helsinki. The Ethics Committees approved all experiments and procedures. All parents or guardians provided written informed consent and the children gave their assent.

### 2.2. Anthropometric and Biochemical Measurements

Anamnesis and physical examination, including the evaluation of sexual maturity according to Tanner’s five-stage scale, were assessed. Body weight, height and waist circumference (WC) were measured using standardized procedures, and body mass index (BMI) was calculated. The BMI Z-score was further calculated based on Spanish reference standards [16]. Obesity was defined using age- and sex-specific IOTF’s BMI cut-off points [17]. Blood pressure was measured three times by the same examiner using an electronic manometer (Omrom, M6 AC) and following international recommendations [18]. 

Blood samples were collected in overnight fasting conditions, centrifuged, and stored at −80 °C. Routine blood tests were conducted at the general laboratory of each participating hospital in an automatic analyzer (Roche-Hitachi Modular P and D Autoanalyzer; Roche Laboratory Systems, Mannheim, Germany) and plasma insulin was analyzed by radioimmunoassay. Insulin resistance (IR) was calculated using the homeostatic model assessment of IR (HOMA-IR). 

Plasma adipokines, inflammation, and cardiovascular risk biomarkers were analyzed using XMap technology (Luminex Corporation, Austin, TX) and human monoclonal antibodies (Milliplex Map Kit; Millipore, Billerica, MA), as previously [19].

### 2.3. Physical Activity Evaluation

PA was objectively evaluated using ActiGraph GT3X+ accelerometers (ActiGraph; Pensacola, FL, USA). Accelerometry is based on measuring the accelerations of the segment of the body where the monitor is connected, usually the right iliac crest, to obtain a more reliable measurement of movement. These accelerometers are able to collect raw acceleration data at sampling frequencies up to 100 Hz over a dynamic range of +6 g. Parents and children were instructed to wear the ActiGraph 24 h per day, 7 days, on the iliac crest on the right hip with an elastic belt. They could be removed only during shower or nocturnal rest (if the instrument caused discomfort during sleep). 

Data were collected at a frequency of 30 Hz. Each device was programmed for time-sampling intervals (epochs) of 15 s. During raw data processing, a minimum of 8 h of monitoring per day for at least 3 days, including at least 1 weekend day was considered acceptable for the evaluation of PA and sedentary time [20]. In addition, two rules were used to exclude low-quality records: (a) All negative counts were replaced by missing data code, and (b) periods of 20 min or more of consecutive zero counts were replaced by missing data code prior to downstream analysis. Unavailability of valid data, non-compliance with the minimum number of hours set or if there was not enough time on valid days during the week or weekend were exclusion criteria for this analysis. The output generated by the ActiGraph GT3X+ included the total volume of PA and the cut-off points of PA intensity recommended by Evenson et al. [20] where sedentary was defined as ≤100 counts per minute (CPM), light PA was defined as 100–2296 CPM, moderate PA was defined as 2296–4012 CPM, and vigorous PA was defined as ≥4012 CPM.

### 2.4. Lifestyle Habits, Screen Time, Sleep Time, and Sport Practice Evaluation

Lifestyle habits were evaluated with questionnaires composed of 54 factors, that were completed by parents or children with help of the researcher. Some of the questions were related to electronic devices, screen time or sport practice based on the National Institute of Child Health and Human Development-validated questionnaire and the screen time-based sedentary behavior questionnaire from the HELENA study [21,22]. All interviews with children were conducted during school time. The average times spent using television, computer or internet, videogames, mobiles, or tablets and the total screen time per day were estimated, for both week and weekend days, alongside time spent sleeping. 

Regarding the habits of PA, the International Physical Activity Questionnaire (IPAQ) [23] and the questionnaire based on sedentary behaviors from the HELENA study [22] were used. Information about different patterns of PA was obtained (time on foot, sports club membership with the specified type of exercise and intensity practiced or different screen-times). Sports that were frequently reported were analyzed individually; less frequent sports were grouped as “others”.

### 2.5. Eating Habits and Mediterranean Diet Adherence

The Krece Plus test, measures the adequacy of the food intake in relation to the Mediterranean diet (considered nutritionally correct). Regarding the score of the test, the child’s diet remains classified as low quality (score < 5.5), regular (could improve) (score = 5.5–8.5), and optimal (score > 8.5). This test has been validated in the Spanish population [24].

### 2.6. Statistical Analysis

The sample size estimation was calculated for the GENOBOX study, based on the principal metabolic risk factors for cardiovascular disease associated with obesity. The calculation of the sample size was carried out for a 95% degree of confidence (type I error alpha = 0.05) and a power of 80% (beta error = 0.20) according to the estimation equation of n by comparison of two proportions of one variable in two independent groups. The sample size under these conditions was raised to a total of 300 to be sure that significant differences could be found for a minimal difference of 20% in each parameter between children with obesity and normal weight. All continuous variables were tested for normality using the Shapiro–Wilk and Kolmogorov test, and all were transformed through natural log, or square root or rank-based inverse normal transformation. Heteroskedasticity between experimental groups was explored with the Levene test. One-way ANOVA and the Kruskal–Wallis test were employed to assess group differences in measurements according to standard statistical assumptions. Pairwise t-tests, pairwise Mann–Whitney U-tests and Dunn tests were applied conveniently as post-hoc analyses, adjusted for BMI Z-score and age, to determine which experimental groups differed from each other. Values in descriptive tables and results are expressed as means and standard deviations or mean relative differences (Δ). Variables related to lifestyle, screen time and sport practice are expressed by frequency percentages of affirmative answers. IBM SPSS Statistics version 22 was used for these analyses.

Clustering is an unsupervised learning technique for finding natural groupings of observations (i.e., clusters) based on the inherent structure within a dataset, useful for reducing highly multivariate datasets. For the identification of cluster subgroups, PA measurements were used as the only input variables. Cluster analyses were performed in R v3.4.4 [25] using the packages “tidyverse”, “cluster”, “factoextra”, “dendextend”, “purr”, and “NbClust”. For both the clustering analysis and for the visual representation in polar line charts, all variables were centered and scaled given the variation in means, variances, and units among them [26]. For that purpose, we employed the scale function available in the R base package. Centering was done by subtracting the variable means (omitting non-available data), and scaling was performed by dividing the (centered) columns by their standard deviations.

Hopkins statistics were applied iteratively, using a threshold of 0.5, before considering the dataset as significantly clusterable (obtained H = 0.82). The cluster analysis was carried out by applying both types of hierarchical clustering available methods (agglomerative and divisive) [27] and by applying Ward’s approach based on Euclidean distances [28]. The agglomerative coefficient obtained using this last method was 0.99. Results are presented according to Ward’s approach. The optimal number of clusters was investigated using the elbow method (k = 3), the Ratkowsky approach (k = 4) and the Scott index (k = 4). Finally, the number of clusters was selected by visual inspection of data (k = 3) (cluster 1, cluster 2, cluster 3). Stability of the cluster solutions was assessed using the cophenetic correlation coefficient (c = 0.35).

Once the clusters were generated using PA measurements, radar charts were generated using the final cluster solution, including all anthropometric and biochemical measurements. 

Since cluster analysis requires there to be no missing data within the study population, all measurements with missing values were preprocessed before clustering by applying the multiple imputations by chained equations (MICE) approach. Briefly, a recursive partitioning technique named classification and regression trees (CART) to infer missing values at the individual level was performed. This method was chosen due to its ability to automatically incorporate nonlinear relations like interaction effects during the imputation process; therefore it is an appropriate technique for the imputation of missing biological data [29].

## 3. Results

**Figure 1:**
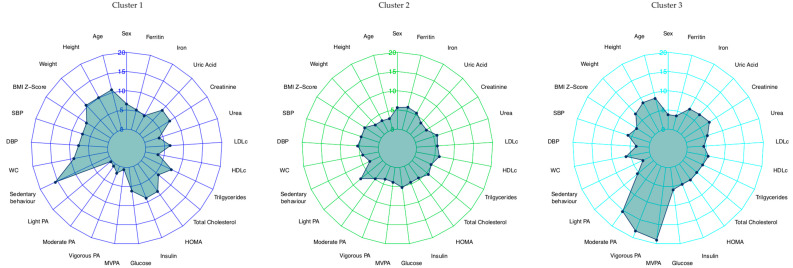
Cluster characteristics based on the measurement of physical activity (cluster 1, cluster 2, cluster 3). DBP: diastolic blood pressure; HOMA-IR: homeostatic model assessment insulin resistance; HDL-c: high-density lipoprotein cholesterol; LDL-c: low-density lipoprotein cholesterol; MVPA: moderate–vigorous physical activity; PA: physical activity; SBP: systolic blood pressure; TAG: triacylglycerides; WC: waist circumference.

Three clusters were established for this study, after some of them were excluded mainly due to lack of accelerometry data, with 489 children selected in total (51.5% female). Radar Chart analysis suggested that the MVPA minutes indicator was adequate to describe the clusters. Cluster 1 (C1; physically inactive) was characterized by low PA levels, and included100 children, Cluster 3 (C3; physically active) was characterized by 73 children with high PA levels, and Cluster 2 (C2; middle group) comprised 294 children between C1 and C3. A total of 22 subjects were excluded due to incomplete data. Figure 1 shows the specific characteristics of each cluster.

In relation to the PA measure, the wear time for accelerometer were similar between groups to be compared, and by intensity, children in C3 had the highest average daily minutes of moderate PA (MPA), vigorous PA (VPA) and MVPA compared with children in C1 and C2. On the other hand, sedentary time was highest in C1 children compared to children in C2 and C3 (Table S1). C1 children had a higher mean age in comparison with C2 children.

In the total sample, there were 243 children with obesity, 108 with overweight and 147with normal-weight. Higher BMI Z-scores and WC values were observed in C1 compared with C2, with lower values observed in C2 compared to C3 for WC. Systolic blood pressure was greater in C1 compared to the other clusters, while diastolic blood pressure was lower in C3 compared to C1 (Table 1). 

C1 presented higher insulin, HOMA, triacylglycerides (TAG) and uric acid levels compared to the other clusters (Table 1).

### 3.1. Lifestyle Habits and Sport Practice

Significant differences were observed between the clusters regarding time spent walking to school, where children in C3 spent more time performing this activity in comparison to those in C1 and C2. Children in C3 spent less time doing homework compared to C1, and children in C1 performed less housework compared to children in the other clusters (Table 2).

The sports most frequently performed by participants in the study were football (24.6%), basketball or volleyball (9.2%), swimming (6.8%), dancing (5.6%), racket sports (3.9%), martial arts (3.3%), skating (2.4%), fitness (1.2%), hiking or running (1.2%), cycling (0.6%), and others (3.6%). A total of 10.1%, of children practiced more than one sport, but 27.6% did not practice any sport. Highest times spent performing MVPA (exceeding 60 min daily) were associated with team sports, such as football, basketball, and volleyball, or some individual sports, such as cycling (Table 2). Children who practiced football generally performed more daily minutes of MVPA compared to children who did not play any sport (Δ = 10.8 ± 3.8 min; *P* = 0.005), but also to those children who swam (Δ = 17.7 ± 6.8 min; *P* = 0.01) or skated (Δ = 24.3 ± 10.7 min; *P* = 0.024).

Over half (55.8%) of C3 children were members of a sport club, whereas only 36.6% of children in C1 practiced sport (*P* = 0.025). Team sports like football (36.5%), basketball and volleyball (12.7%) were common in C3 children, whereas for example racket sports were predominant in C1 (11.1%). 

### 3.2. Screens, Electronic Devices, and Sleep

C3 children spent more time using mobile phones or tablets than C2 children. A significant difference was observed between television time and time spent using devices (P < 0.05). C1 children spent more time using electronic devices in their rooms compared with C2 and C3 children, especially televisions and computers. A higher percentage of C1 children had their own mobile phone compared to children in C2 and C3, with a highly significant difference. No significant differences were observed between clusters in the meantime per day or week spent using screens or electronic devices (Table 3). 

In relation to time spent sleeping on weekdays, C1 children slept less than C2 (P < 0.001) and C3 (*P* = 0.031) children. No differences were found between clusters on weekend days (Table 3).

### 3.3. Eating Habits and Mediterranean Diet Adherence 

A subsample of 158 children was selected to analyze eating habits. The percentages of children that were included in each of the three diet quality levels for the Krece Plus questionnaire are presented in Table 4. No differences were observed in the percentage of children in relation with the adequacy of the food intake to the Mediterranean diet between the different clusters. Most participants exhibited low or moderate scores.

## 4. Discussion

This study showed that lifestyle patterns, PA or sedentary behavior, use of electronic devices, screen time, sleep, and metabolic status in recruited children are clustered into three differential groups. Sedentary patterns were more often seen in older children, who had higher BMI Z-scores, higher systolic blood pressure and other biochemical metabolic risk factors [30] compared to children who were in an average pattern of PA, i.e., between sedentary and active. Less use of electronic devices and less screen time were observed in the most active children, as well as greater participation in sport clubs and more time spent practicing MVPA during the day. In fact, the recommended 60 minutes of PA to promote metabolic health [31] was exceeded by children in the active group, who were associated to a lower metabolic risk according to their measured biomarkers.

Cluster analysis was used by other authors to identify different profiles depending on behavior patterns, lifestyle, PA, or dietary habits [32,33,34,35]. A review [36] including 18studies in children and adolescents based on the influence of PA, sedentary behavior and diet on health, observed that these factors were grouped in children in healthy and unhealthy patterns, although some behaviors were shared. Specifically, groups with high levels of sedentarism were frequently observed. In addition, previous studies [34,35] obtained similar clusters as to the ones found in the present study, also based on objectively measured PA. One study reported that the group who spent the most time performing MVPA and the least in a sedentary state was associated with a healthier weight status [34]. Similarly, previous studies [11,37] observed that older children exhibited an increased sedentary lifestyle and practiced less PA. Low levels of PA and high time spent in sedentary behavior were also associated with an overweight state [38].

In the present study, most of the sample was grouped into C2, with a minority being physically active. C2 children spent more time performing MVPA on average than those in the sedentary group, with no differences observed regarding VPA between these two groups. However, the active group was characterized by more MVPA and especially more VPA, without showing differences in sedentary time and light PA, compared to active C2 children. This finding reinforced the importance of MVPA in achieving a physically active lifestyle, as well as the associations previously found in this other clustering work in school-aged children [34].

Sedentary behavior was independently and positively associated with a worse state of health [39,40]. In the present study, C3 children spent the least amount of time in a sedentary state; therefore, future interventions should focus on encouraging a more active lifestyle.

Children and adolescents spend more time participating in sedentary activities now than a decade ago due to the increasing use of screen-based electronic devices (e.g., smartphones, laptops) and the wide accessibility of the Internet [4]. In our study, an association with a high usage time of technological devices was observed, particularly in sedentary children with easy access to devices in their own room or through a personal mobile phone. Therefore, restricting or moderating access to electronic devices in children could promote a more active lifestyle pattern. 

Strong associations exist between sedentary behavior based on screen time and health problems [5,6]. A systematic review [5] reported positive associations between screen time and sleep problems, musculoskeletal pain, and depression. They also identified negative associations between screen time and PA/physical condition, psychological well-being, and social support. However, no relationship was observed between sedentary behavior as a result of use of electronic devices and diet quality. Our data supported that children with a sedentary pattern showed higher electronic device use and screen time and had generally, higher BMIs. 

The results of the study showed how the participants, regardless of the cluster in which they were grouped, approached, or surpassed two hours of daily screen time during the week, exceeding the recommendation for children to spend a maximum of one hour per day performing screen-based sedentary activities [41,42]. A recent report on Spanish children and adolescents described that children exceeded these recommendations by more than four and a half hours during the weekend, in agreement with our study [43]. Due to the development of technology and early access to these advances, screen time patterns recommendations for children should be reviewed periodically [5].

Beyond the association between these factors and sedentarism, other relationships were found in the most active children, who invested more time in daily activities, with more exercise, such as walking to school, or doing household chores. Moreover, active children spent less time doing school tasks or other activities involving sedentary time.

Except for children in the sedentary group who spent less time sleeping than the others, time spent sleeping was generally adequate, exceeding the nine hours of sleep recommended in childhood [41]. However, in addition to duration, sleep quality should be further investigated.

A positive relationship has been reported between participation in extracurricular sports activities and better child development [44,45,46]. In addition, sports practice from an early age is associated with an active style in adult life, as shown by a longitudinal study with a follow-up of 21 years [47]. In the present work, a higher percentage of children were members of a sport club (55.8%) in the active group versus sedentary children (36.6%). Taking into account that the PA derived from some sports could not be well quantified, such as swimming due to the inability to wear the accelerometer into the water, active children also showed more time spent performing MVPA in collective sports, such as football, basketball or volleyball, than those in the sedentary cluster. The choice of sport, along with time spent practicing should be considered to promote an active lifestyle. Moreover, the dimensions in which sport activities are carried out is a determining factor, since they influence the number of different activities that children perform, as well as the intensity, duration (session time and number of sessions in months or years), motivation, adherence and type of commitment (e.g., social, emotional, physical, cognitive) [48,49,50]. In fact, team sports provide further benefits in psychological and social health in children [51]. Therefore, engaging in sports as extracurricular activities seems to result in a better state of physical and self-perceived health [48].

However, an active lifestyle must be combined with adherence to a healthy diet [51,52]. Indeed, parameters of PA practice measured by the PAQ-C questionnaire have been associated with a healthy diet [53]. Higher subjectively measured MVPA practice in children and adolescents was associated with higher consumption of fiber-rich carbohydrates and monounsaturated and polyunsaturated fatty acids, better management of techniques to control stress and higher quality of sleep, especially in those who practiced more VPA [7]. In a Spanish study of 631 physically active students, good physical condition and optimal adherence to the Mediterranean diet was generally exhibited [52]. However, the lack of objective assessment of PA when interpreting literature results could be a limitation to establish its relationship with lifestyle habits. PA and sedentary behavior are usually measured using different methods or are expressed in different units. Therefore, to quantify PA objectively, accelerometry is the most reliable method [54], with ActiGraph® being the system most commonly used by researchers [55].

In contrast to previous works, the clustering in this study included a high number of variables such as metabolic risk markers, which are not usually included in lifestyle clustering studies [36]. Furthermore, our study also performed an objective measurement of PA through accelerometry, but we did not find differences in Mediterranean diet adherence between the groups, with the majority of the children with a regular quality, although this evaluation was achieved only in a subsample of children in this work. Therefore, the influence of diet quality on the level of active lifestyle should be investigated in the future.

## 5. Conclusions

The present work identified a cluster of active children with a healthier lifestyle pattern encompassing a minority of the studied population. To promote the activity of children during the growth stage, electronic and screen devices should be kept away from bedrooms in order to stimulate greater collaboration in the household and to increase exercise during extracurricular time.

## Figures and Tables

**Table 1:** Characteristics, anthropometry, time spent in sedentary behavior and physical activity intensity, and metabolic markers, by clusters.

Variables	Cluster 1 (*n* = 100)	Cluster 2 (*n* = 294)	Cluster 3 (*n* = 73)	*P*(ANCOVA)
**Characteristics of sample and anthropometric measurements**				
Age (years)	13.2 ± 0.84^(a)^	10.2 ± 2.24^(b)^	11 ± 2.2^(a)^	**0.027**
Height (cm)	151 ± 12	140 ± 14	149 ± 14	0.355
Weight (kg)	60.8 ± 19^(a)^	47.1 ± 17.8^(b)^	54.4 ± 20^(ab)^	0.074
BMI Z-score	2.15 ± 1.65^(a)^	1.75 ± 1.84^(b)^	1.70 ± 1.68^(ab)^	0.094
Normal-weight (n)	20 (19.6%)	90 (30.3%)	22 (31.2%)	0.144 ^†^
Over-weight (n)	25 (24.5 %)	61 (20.%)	21 (27.3%)	
Obesity (n)	55 (55.9 %)	143 (49.4%)	30 (41.6%)	
SBP (mmHg)	112.3 ± 14.5^(a)^	108 ± 13.1^(b)^	108.8 ± 13.9^(ac)^	**0.048**
DBP (mmHg)	67.1 ± 10.2^(a)^	65.6 ± 8.9^(ab)^	63.9 ±9.9^(b)^	0.113
WC (cm)	85.1 ± 16.1^(a)^	77.1 ± 15.8^(b)^	80.5± 17.1^(ab)^	0.393
**Time spent in sedentary behavior and physical activity by intensity (minutes/day)**				
Accelerometer wear time on hip (days)	4.72 ± 0.80	4.75 ± 0.69	4.63 ± 0.74	0.486
Sedentary behavior	546.5 ± 62.3^(a)^	439 ± 58.9^(b)^	433.5 ± 78.5^(bc)^	**<0.001**
Light PA	213.3 ± 56^(a)^	260.2 ± 53.1^(b)^	246.7 ± 73.9^(bc)^	**<0.001**
Moderate PA	30.3 ± 11.2^(a)^	36.4 ± 11.4^(b)^	52.2 ± 15.8^(c)^	**<0.001**
Vigorous PA	10.1 ± 7.5^(a)^	12.4 ± 6.8^(ac)^	33.5 ± 20.4^(b)^	**<0.001**
MVPA	40.4 ± 17.2^(a)^	48.8 ± 16.1^(b)^	85.7 ± 25.3^(c)^	**<0.001**
**Metabolic markers**				
Glucose (mg/dL)	86.48 ± 7.94	85.75 ± 7.35	86.14 ± 7.86	0.983
Insulin (mU/L)	15.17 ± 9.4^(a)^	10.58 ± 9^(b)^	11.02 ± 7.64^(b)^	**0.016**
HOMA-IR	3.27 ± 2.08^(a)^	2.28 ± 2^(b)^	2.38 ± 1.69^(b)^	**0.020**
Cholesterol (mg/dL)	164.81 ± 32.59	164.21 ± 28.14	160.84 ± 23.58	0.751
TAG (mg/dL)	78.63 ± 34.55^(a)^	68.60 ± 33.36^(b)^	65.82 ± 38.69^(b)^	**0.001**
HDL-c (mg/dL)	49.66 ± 13.01	53.63 ± 15.24	53 ± 13.97	0.103
LDL-c (mg/dL)	97.39 ± 29.02	94.71 ± 25.98	90.85 ± 21.08	0.263
Urea (mg/dL)	29 ± 9.33	30.46 ± 7.95	30.54 ± 7.46	0.313
Creatinine (mg/dL)	0.58 ± 0.10	0.54 ± 0.11	0.58 ± 0.13	0.601
Uric Acid (mg/dL)	4.8 ± 1.09^(a)^	4.37 ± 1.12^(b)^	4.63 ± 1^(ab)^	**0.004**
Iron (ug/dL)	82.28 ± 32.26	81.52 ± 33.25	85.89 ± 30.27	0.596
Ferritin (ng)	50.59 ± 25.60	55.33 ± 40.48	47.13 ± 24.39	0.171

DBP: diastolic blood pressure; HOMA-IR: homeostatic model assessment insulin resistance; HDL-c: high-density lipoprotein cholesterol; LDL-c: low-density lipoprotein cholesterol; MVPA: moderate and vigorous physical activity; PA: physical activity; SBP: systolic blood pressure; TAG: triacylglycerides; WC: waist circumference. Mean ± standard deviation. ANCOVA adjusted for age, or age and BMI as indicated. No matching superscript letters (a, b, c) indicate significant differences by pairwise Mann–Whitney U-tests and Dunn post-hoc test (*P* < 0.05). ^†^ *P*-value significance was obtained after chi-squared analysis test, in bold.

**Table 2:** Spent in lifestyle habits, and percentage of children practicing extracurricular sport activities with global time for of moderate to vigorous physical activity (MVPA) by clusters.

Variables		Cluster 1 (*n* = 100)	Cluster 2 (*n* = 294)	Cluster 3 (*n* = 73)	*P* (ANCOVA)
**Time in hours**					
Physical education class		3.1 ± 0.4	3.2 ± 0.5	3.1± 0.4	0.339
Walking to school		6.1 ± 5.8^(a)^	4.8 ± 5.6^(^^ab)^	9.2 ± 9.8^(^^c)^	**<0.001**
Doing homework		3.6 ± 1^(a)^	3.3 ± 1^(ab)^	3.2 ± 1^(b)^	**0.039**
Doing week housework		1.6 ± 2.1^(a)^	2.5 ± 2.9^(ab)^	2.6 ± 2.3^(c)^	**0.017**
Doing weekend housework		1.3 ± 2	1.5 ± 2.2	1.1 ± 1.5	0.142
PA in family along week		1.4 ± 2.4	1.8 ± 4.1	1.9 ± 3.8	0.816
PA in family along weekend		1.8 ± 1.5	1.9 ± 1.6	2 ± 1.6	0.722
Exercising in a sports club		2.2 ± 2	2.3 ± 2.2	2.5 ± 2.9	0.872
**Extracurricular sports**	**MVPA (min)**	**Percentages of children**			***P*^†^**
Football	64.4 ± 2.9 *	18.5	22.7	36.5	**0.012**
Basketball or volleyball	61.6 ± 4.9	7.4	8.6	12.7	
Swimming	46.7 ± 6.2	9.3	7.3	3.2	
Dancing	48 ± 8.7	5.6	6.4	3.2	
Racket sports	58.6 ± 7.3	11.1	2.7	1.63	
Martial arts	48.8 ± 8.2	0	4.1	3.2	
Skating	40 ± 10.3	7.4	1.8	0	
Fitness	55.5 ± 16.3	0	1.8	0	
Hiking or running	59 ± 11.6	0	1.8	0	
Cycling	64.8 ± 16.3	0	0.5	1.6	
Others	54.4 ± 7	5.6	2.3	6.3	
More than one sport	52.4 ± 5.8	5.6	13.2	3.2	
None	53.6 ± 2.6	29.6	26.8	28.6	

PA: physical activity; MVPA: moderate and vigorous physical activity. ANCOVA adjusted for age or, age and BMI as indicated. No matching superscript letters (a, b, c) indicate significant differences by pairwise Mann–Whitney U-tests and Dunn post-hoc test (*P* < 0.05). ^†^ *P*-value significance was obtained after chi-squared analysis test. * Significative differences were only observed in MVPA minutes spent playing football compared with swimming, skating and no sport (*P* ≤ 0.05, in bold).

**Table 3:** Use of electronic devices and screen time by clustering.

Variables	Cluster 1 (*n* = 100)	Cluster 2 (*n* = 294)	Cluster 3 (*n* = 73)	
**In possession of (%)**				***P*^†^**
TV in the room	47.1	33.7	28.9	**0.02**
TV at home	100	92.8	98.7	**0.004**
Computer in the room	41.2	23.9	31.6	**0.003**
Computer at home	75.5	80.4	77.6	0.55
Internet in the room	43.1	24	31.6	**<0.001**
Internet at home	62.7	71.8	68.4	0.224
Sound system in the room	23.5	26.6	25	0.813
Sound system home	79.4	76.3	81	0.646
Videogames in the room	38.2	28.3	31.6	0.169
Videogames at home	72.5	68.2	67.1	0.664
Own mobile phone	53.9	23.3	44.7	<0.001
Home mobile phone	74.5	64.6	68.4	0.176
**Screen TV in time zones (%)**			***P*^†^**
Between 6 and 9 am	23.8	36.7	23.4	**0.012**
Between 9 and 12 am	3	10.8	5.2	**0.027**
Between 12 and 3 pm	38.6	37.4	33.8	0.788
Between 3 and 6 pm	47.5	36.7	40.3	0.155
Between 6 and 9 pm	62.4	6.3	58.3	0.393
Between 9 and 12 pm	70.3	57.7	59.7	0.079
**Time of screen (hours)**				***P* (ANCOVA)**
Daily average on week	1.9 ± 0.5	1.7 ± 0.5	1.9 ± 0.6	0.399
Daily average on weekend	5.7 ± 1.4	5.5 ± 1.5	5.8 ± 1.6	0.761
Watching TV on week	3.8 ± 1.2	3.5 ± 1.1	3.8 ± 1.2	0.504
Watching TV on weekend	4.5 ± 1.3	4.5 ± 1.2	4.5 ± 1.4	0.965
Using of computer or internet on week	2.2 ± 1.2	2 ± 1.1	2.2 ± 1.2	0.594
Using of computer or internet on weekend	2.5 ± 1.4	2.5 ± 1.4	2.5 ± 1.7	0.899
Playing videogames on week	1.6 ± 1	1.6 ± 1	1.5 ± 1	0.581
Playing videogames on weekend	2.2 ± 1.4	2.4 ± 1.4	2.2 ± 1.4	0.384
Using mobile phone or tablet on week	1.9 ± 1.4^(a)^	1.4 ± 0.9^(ab)^	2 ± 1.6^(ac)^	**0.005**

TV: television; Mean ± standard deviation. ANCOVA adjusted for age or, age and BMI as indicated. No matching superscript letters (a, b, c) indicate significant differences by pairwise Mann–Whitney U-tests and Dunn post-hoc test (*P* < 0.05). ^†^ *P*-value significance was obtained after chi-squared analysis test, in bold.

**Table 4:** Percentage of children in each cluster with adequacy of the food intake to the Mediterranean diet measured by scores based in Krece Plus questionnaire, by clusters.

Levels of Quality of Diet—Kreceplus	Cluster 1 (*n* = 27)	Cluster 2 (*n* = 96)	Cluster 3 (*n* = 35)	Total sample (mean)	*P*
Low quality (score < 5.5)	13.3	63.3	23.3	19	0.551
Regular quality (score = 5.5–8.5)	16	59.3	24.7	51.3	
Optimal quality (score > 8.5)	21.3	61.7	17	29.7	
Total Score	7.5± 2.1	7.2 ± 2	7.2± 2	7.3± 2	

*P*-value significance was obtained after chi-squared analysis test.

## Supplementary Materials

The following are available online at https://www.mdpi.com/2072-6643/12/5/1288/s1, Table S1: Statistical transformed data of variables showed in Table S1, that not presented a normal distribution.
