# Supplementary material for: Cluster Analysis of Physical Activity Patterns, and Relationship with Sedentary Behavior and Healthy Lifestyles in Prepubertal Children: Genobox Cohort
Source: Nutrients. 2020 May 1;12(5):1288. doi: 10.3390/nu12051288 (PMC7282254; doi:10.3390/nu12051288)
Supplement: Supplementary file 1 [file nutrients-12-01288-s001.pdf]

Supplementary Table S1. Statistical transformed data of variables showed in table S1, that not presented a normal distribution.

Variables	Cluster 1 (n=100)	Cluster 2 (n=294)	Cluster 3 (n=73)	*P*(ANCOVA)
Characteristics of sample and anthropometric measurements				
Age (years)	3.37± 0.34[(a)]	3.13 ± 0.19[(b)]	3.31 ± 0.39[(a)]	**0.027**
Height (cm)	0.41± 0.08	0.34 ± 0.1	0.39 ± 0.1	0.355
Weight (kg)	7.70± 1.22[(a)]	6.74 ± 1.28[(b)]	7.25 ± 1.34[(ab)]	0.074
DBP (mmHg)	4.19 ± 0.15[(a)]	4.17 ± 0.13[(ab)]	4.15 ± 0.15[(b)]	0.113
WC (cm)	4.43 ± 0.19[(a)]	4.32 ± 0.21[(b)]	4.37± 0.21[(ab)]	0.393
Time physical activity intensity and sedentary behavior (minutes/day)				
Moderate PA	3.34 ± 0.37[(a)]	3.53 ± 0.38[(b)]	3.90 ± 0.34[(c)]	**<0.001**
Vigorous PA	2.06 ± 0.75[(a)]	2.34 ± 0.65[(b)]	3.37 ± 0.54[(c)]	**<0.001**
MVPA	3.61 ± 0.42[(a)]	3.82 ± 0.40[(b)]	4.41 ± 0.29[(c)]	**<0.001**
Metabolic markers				
Glucose (mg/dL)	4.45 ± 0.09	4.45 ± 0.09	4.45 ± 0.09	0.983
Insulin (mU/L)	3.73 ± 1.12[(a)]	3.02 ± 1.06[(b)]	3.14 ± 1.19[(b)]	**0.016**
HOMA-IR	1.73 ± 0.53[(a)]	1.40 ± 0.51[(b)]	1.45 ± 0.52[(b)]	**0.020**
TAG (mg/dL)	4.28 ± 0.42[(a)]	4.12 ± 0.45[(b)]	4.06 ± 0.48[(b)]	**0.001**
HDL-c (mg/dL)	3.87 ± 0.26	3.93 ± 0.30	3.94 ± 0.26	0.103
LDL-c (mg/dL)	9.75 ± 1.50	9.64 ± 1.34	9.46 ± 1.12	0.263
Urea (mg/dL)	3.32 ± 0.28	3.38 ± 0.25	3.39 ± 0.24	0.313
Ferritin (ng)	7.02 ± 1.72	7.12 ± 2.29	6.63 ± 1.72	0.171

Variables transformed by means of natural log: Height; Glucose; Urea; DBP: diastolic blood pressure; HDL: high-density lipoprotein; TAG: triacylglycerides; WC: waist circumference; MVPA: moderate and vigorous physical activity; PA: physical activity (moderate, vigorous, and moderate-vigorous). Variables transformed by means of square root: Age; weight; Insulin; HOMA-IR; Ferritin; LDL: low-density lipoprotein. Mean ± standard deviation. ANCOVA adjusted by age or, age and BMI as indicated. No matching superscript letters (a, b, c) indicate significant differences by Dunn post-hoc test ($P< 0.05$).
